# Beyond bacterial defences: the role of lysozyme in cancer

**DOI:** 10.1002/ctm2.70575

**Published:** 2026-02-01

**Authors:** Lei Wang, Qian Dong, Fuchu He, Zhiwen Gu, Aihua Sun

**Affiliations:** ^1^ College of Chemistry and Life Science Beijing University of Technology Beijing China; ^2^ State Key Laboratory of Medical Proteomics, Beijing Proteome Research Center, National Center for Protein Sciences (Beijing), Research Unit of Proteomics‐Driven Cancer Precision Medicine (Chinese Academy of Medical Sciences) Beijing Institute of Lifeomics Beijing China; ^3^ Department of Laboratory Medicine Peking University Third Hospital Beijing China; ^4^ Core Unit of National Clinical Research Center for Laboratory Medicine Peking University Third Hospital Beijing China

**Keywords:** biomarker, cancer, lysozyme, LYZ, multi‐omics, therapeutic target

## Abstract

Lysozyme (LYZ) is a naturally occurring antimicrobial protein first discovered in the 1920s. As a key component of innate immunity, its antimicrobial effects and immunomodulatory functions in bacterial defence have been extensively characterized. However, emerging evidence since the 1950s has revealed its complex involvement in tumour progression, with conflicting reports of both tumour‐suppressive and tumour‐promoting effects across different cancer types. A critical knowledge gap remains in understanding the mechanistic basis for this duality, exacerbated by reliance on single‐omics approaches and small cohorts in previous studies. This review focuses on mammalian C‐type LYZ (referred to as LYZ hereafter unless specified) and integrates multi‐omics data (transcriptomics and proteomics) with clinical and mechanistic research to systematically dissect its dual roles in cancer. By analysing cross‐cancer heterogeneity through multi‐omics perspectives, we emphasize its dual promise as both a prognostic biomarker and an actionable therapeutic target, aiming to provide new insights for precision oncology.

**Key points:**

- LYZ is a multi‐functional secreted factor that encompasses both antibacterial and immunomodulatory functions.
- Emerging evidence highlights its complex role in tumour progression by directly influencing tumour cells and modulating the immune microenvironment.
- LYZ is a promising potential biomarker and therapeutic target in some cancers.

## Graphical abstract

Lysozyme (LYZ) is a multi‐functional secreted factor that encompasses both antibacterial and immunomodulatory functions. Emerging evidence highlights its complex role in tumour progression by directly influencing tumour cells and modulating the immune microenvironment. In addition, LYZ is a promising potential biomarker and therapeutic target in some cancers.

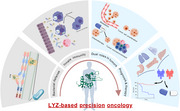

## 1 INTRODUCTION

**FIGURE 1:**
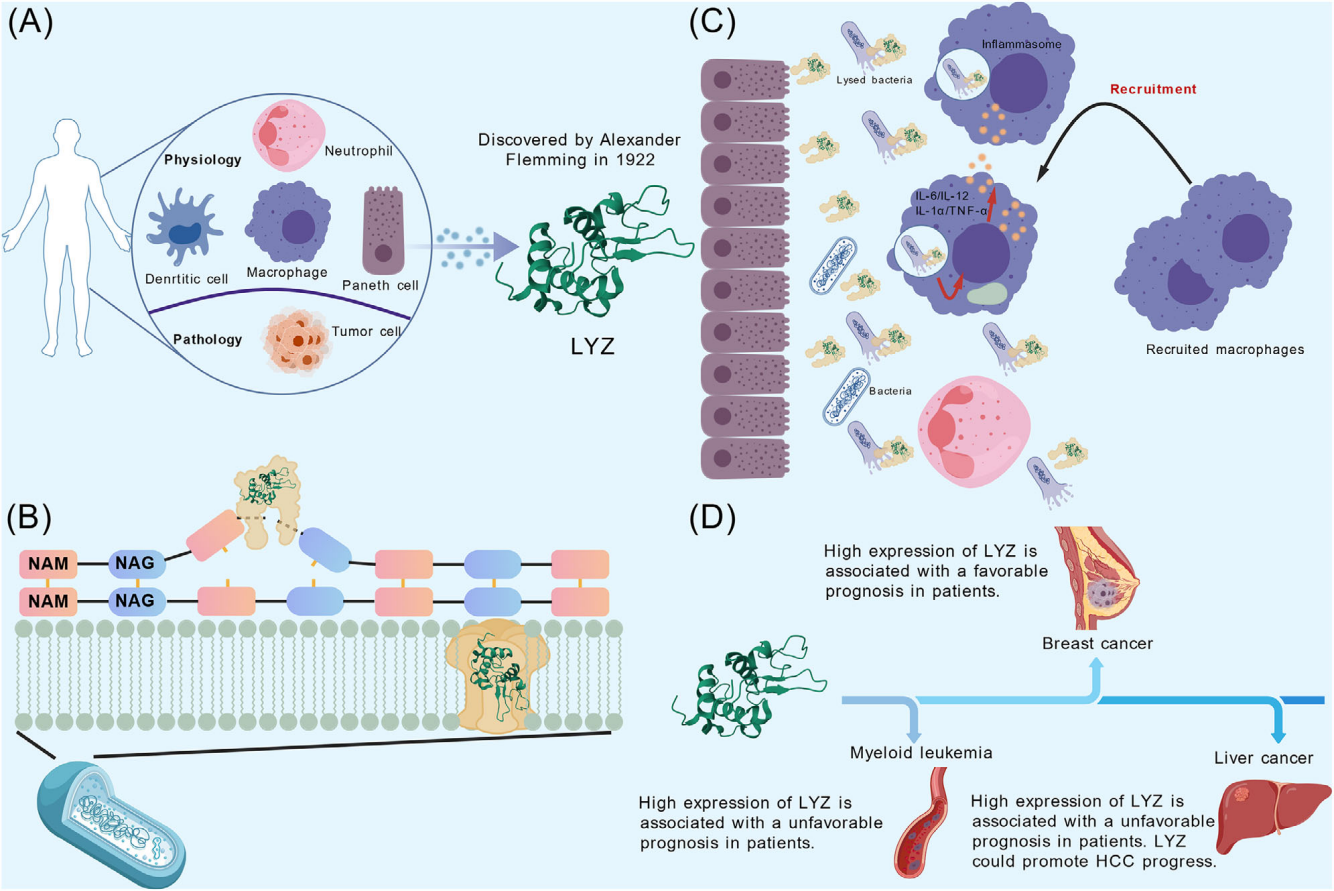
Physiological and pathological expression of lysozyme (LYZ) and its biological function beyond bacteria killing. (A) LYZ is largely expressed by myeloid cells and Paneth cells in mammals, but some malignant tumour cells also abnormally express LYZ. (B) LYZ can hydrolyse bacterial cell walls through two mechanisms, leading to bacterial death. It cleaves the β‐1,4 glycosidic bonds between *N*‐acetylglucosamine (NAG) and *N*‐acetylmuramic acid (NAM) in the peptidoglycan layer. Additionally, due to its cationic properties, LYZ can interact with cell membranes and the negatively charged components, forming pores in the bacterial cell membranes and ultimately leading to bacterial death. (C) At bacterial invasion sites, LYZ secreted by epithelial cells (mainly Paneth cells) or myeloid immune cells can directly hydrolyse bacteria, leading to the release of numerous pathogen‐associated molecular patterns (PAMPs), which in turn recruit more phagocytes to boost the pro‐inflammatory immune response. In addition, macrophages internalize bacteria for LYZ‐mediated degradation within phagosomes, resulting in the release of additional PAMPs to stimulate a strong pro‐inflammatory cytokine response and activate inflammasomes. (D) Unexpected role of LYZ in cancer prognosis and progression for tumours. In lung and breast cancers, LYZ is considered to be associated with tumour suppression. However, in myeloid leukaemia and liver cancer, LYZ has been reported to promote tumour progression and thusly associated with poor prognosis. HCC, hepatocellular carcinoma. This figure was generated using the Generic Diagramming Platform‐BioGDP.^9^

Lysozyme (LYZ) was first discovered in human nasal mucus, tears and saliva by Fleming in 1922 and was named for its bacteriolytic effect.^1^ As a conserved antibacterial protein, it is abundantly expressed in mammalian tissues (e.g. stomach, salivary glands, bone marrow, small intestine, lungs and spleen^2^, ^3^, ^4^) and secretions (e.g. milk, saliva, tears^5^), and is also produced by myeloid cells such as macrophages, neutrophils and dendritic cells^5^ (Figure 1A). Recent studies have progressively revealed LYZ expression in non‐traditional cell types, including neurons,^6^ fibroblasts^7^ and endocardial cells,^8^ which expands the understanding of its biological significance.

Structurally, LYZs possess a universal catalytic function in hydrolysing bacterial peptidoglycan cell walls, with three primary subtypes identified based on amino acid sequences and biochemical properties: C‐type (chicken/conventional), G‐type (goose) and I‐type (invertebrate). Additional types, including phage‐, bacterial‐ and plant‐type LYZs, have since been characterized.^10^ But mammalian C‐type LYZ remains the focus of cancer‐related research due to its widespread expression and immunomodulatory roles. Among them, C‐type and G‐type LYZs are basic proteins with high isoelectric point (pI) values. In contrast, various I‐type LYZs exhibit significant differences in their pIs, which may be related to their distinct functional roles.^11^ The molecular weight of mature C‐type and I‐type LYZ typically ranges from 11 to 15 kDa, while that of G‐type LYZ ranges from 20 to 22 kDa.^12^

**FIGURE 2:**
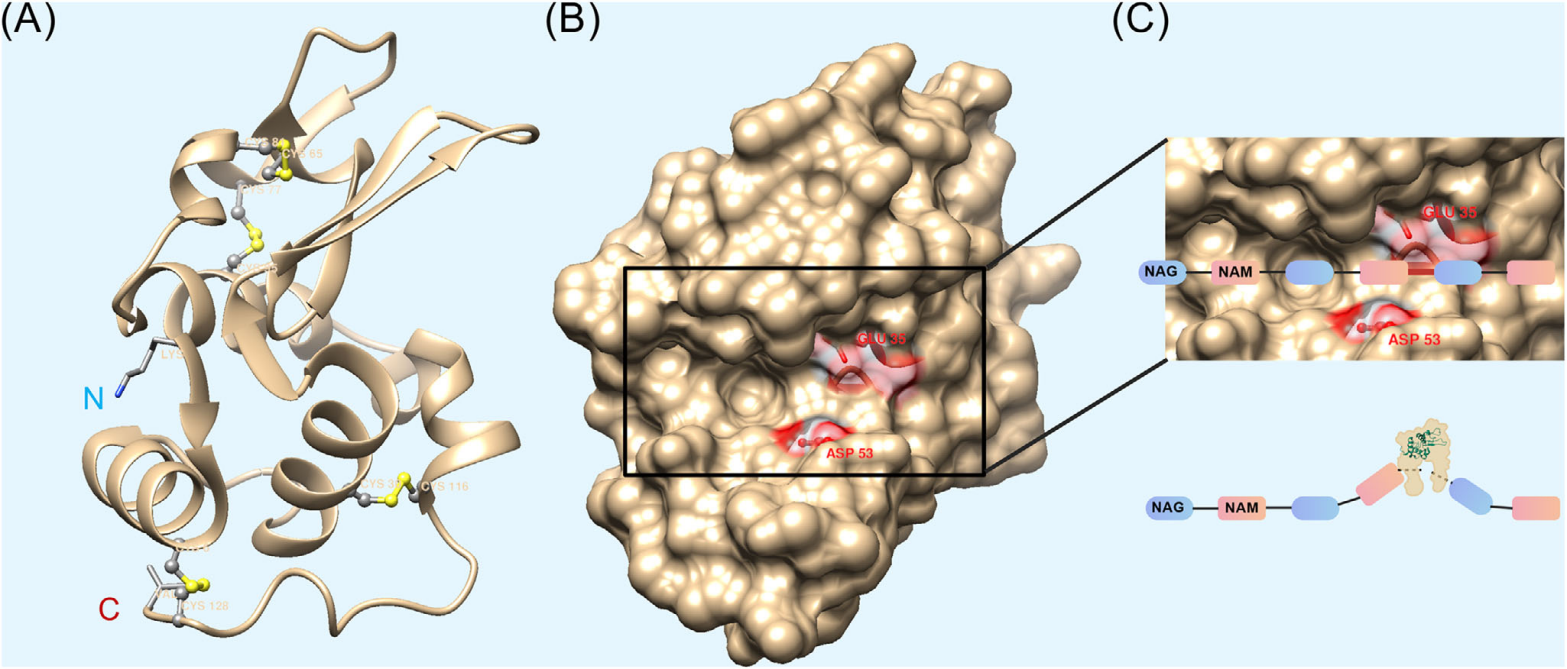
Biochemical and functional characteristics of lysozyme (LYZ). (A) Ribbon diagrams of three‐dimensional structure of human LYZ from the PDB (Protein Data Bank) Database (PDB, entry 133L). LYZ is structurally composed of four α‐helices and three β‐sheets. Its structure contains four disulfide bonds (Cys6‐Cys128, Cys30‐Cys116, Cys65‐Cys81, Cys77‐Cys95). The N‐terminus (Lys) and C‐terminus (Val) of LYZ are labelled in the figure. Grey stick‐and‐ball models represent Cys residues. Yellow stick‐and‐ball models represent the disulfide bonds formed between Cys residues. (B) Space‐filling model of LYZ (PDB, entry 133L). The enzymatic active centre of LYZ comprises two crucial amino acid residues: Glu35 and Asp53. The two amino acid regions are highlighted in red, with their residue positions displayed. (C) The active site cleft of LYZ accommodates six consecutive sugars from peptidoglycan, and specifically cleaves the β‐1,4‐glycosidic bond between the fourth and fifth sugar residues (Indicated by bold red line). The protein structure of LYZ was visualized using UCSF Chimera (version 1.11.2). The three‐dimensional structure of LYZ does not include the signal peptide region. Asp, aspartate; Cys, cysteine; Glu, glutamate; Lys, lysine; NAG, *N*‐acetylglucosamine; NAM, *N*‐acetylmuramic acid; Val, valine. This figure was generated using the Generic Diagramming Platform‐BioGDP.^9^

The human C‐type LYZ will be examined as a model to elaborate on its biochemical profile. The full‐length LYZ polypeptide is 148 amino acids. Following the cleavage of an 18‐residue signal peptide, the mature functional protein comprises 130 amino acids. Although sequence homology is low between human and other C‐type LYZs, their secondary structures are highly conserved. The structure of LYZ, comprised of α and β domains, houses four α‐helices, three β‐sheets and is stabilized by four disulfide bonds^10^ (Figure 2A). LYZ adopts a compact, ellipsoidal structure featuring a prominent cleft capable of accommodating polysaccharide substrates. This structural motif, shared among diverse LYZ types, underscores a high degree of evolutionary conservation. The LYZ catalytic activity is primarily mediated by a pair of key residues: Glu35 and Asp53 within the polypeptide chain, which constitute the core of its active site^13^ (Figure 2B). The active site of LYZ binds the substrate through six complementary sites, accommodating six consecutive sugars. Concurrently, it cleaves the β‐1,4‐glycosidic linkage between the fourth and fifth sugar residues^5^, ^12^ (Figure 2C). This meticulously structured active site ensures remarkable functional stability.

### 1.1 The traditional functions of LYZ

One of the most compelling functions of LYZ is the excellent bactericidal effect. LYZ's canonical bactericidal functions occur via two mechanisms: enzymatic cleavage of β‐1,4 glycosidic linkage between *N*‐acetylmuramic acid (NAM) and *N*‐acetylglucosamine (NAG) in peptidoglycan,^14^, ^15^, ^16^, ^17^, ^18^ and, uniquely in C‐type LYZs, membrane pore formation via interactions with negatively charged components^19^, ^20^ (Figure 1B). The enzymatic activity of LYZ serves as the primary driver for the bactericidal function across different LYZ types. In addition, LYZ shares similar ‘Helix–Loop–Helix’ structural domains and possesses enzymatic active sites centred around glutamate and aspartate residues.^14^ The other mechanism is unique to C‐type LYZ. Due to LYZ's cationic nature, it can interact with negatively charged components in cell membranes,^19^ leading to pore formation in the membranes and ultimately to bacterial death.^20^ Both the isolated Helix–Loop–Helix peptide and the *N*‐terminal helical segment derived from LYZ demonstrate significant bactericidal efficacy against bacteria. These antimicrobial peptides kill bacteria by penetrating the bacterial outer membrane and disrupting membrane potential‐dependent respiration.^21^ In addition, specific modifications to bacterial peptidoglycan components, alterations in their charges and the secretion of inhibitory factors can also impede LYZ activity.^5^

Beyond bacterial defence, LYZ is a cornerstone of innate immunity,^22^ regulating immune cell activation,^23^ inflammatory responses (via pathways like the nucleotide binding oligomerization domain containing 1 [NOD1] receptor/nucleotide binding oligomerization domain containing 2 [NOD2] receptor^24^, ^25^, ^26^ and toll‐like receptors [TLRs]^27^, ^28^, ^29^) and pathogen clearance^5^ (Figure 1C). LYZ plays a dual role in inflammation. On the one hand, its mediated bacterial degradation facilitates phagocyte activation, enhancing the release of bacterial products and the generation of inflammatory mediators. Conversely, LYZ also contributes to the resolution of inflammation. The bacterial killing by LYZ, secreted from epithelial cells and macrophages, leads to the release of pathogen‐associated molecular patterns (PAMPs). These PAMPs subsequently recruit additional phagocytes to clear bacteria and concurrently activate the inflammasome through NOD1/NOD2 receptors and TLRs.^5^ This cascade ultimately enhances the secretion of pro‐inflammatory cytokines, thereby fostering the establishment of a pro‐inflammatory microenvironment. On the other hand, insoluble peptidoglycans in the extracellular environment trigger strong chemotaxis of phagocytes through complement factors such as C3a and C5a. LYZ converts these peptidoglycans into soluble small fragments, which helps reduce the production of these factors and consequently diminishes phagocyte infiltration.^5^, ^30^ This process contributes to the alleviation of inflammatory responses in vivo. LYZ achieves a balanced inflammatory response through its dual regulatory mechanism.

### 1.2 Regulation of LYZ

For decades, research has not only elucidated the traditional functions of LYZ but has also consistently focused on the mechanisms of its regulation. Cumulative evidence from models including chickens, mice and zebrafish has clarified the transcriptional, post‐transcriptional and epigenetic pathways governing LYZ expression and functional activity – revealing a complex, multi‐layered regulatory network.

#### 1.2.1 Transcriptional regulation

Transcriptional control of the *LYZ* gene is tightly modulated by species‐specific cis‐regulatory elements and transcription factors. In chickens, *LYZ* gene expression in the oviduct and macrophages is governed by tissue‐specific cis‐regulatory elements.^31^, ^32^ The chicken LYZ locus contains three enhancers (located at −6.1, −3.9, and −2.7 kb), a hormone response element (−1.9 kb), a silencer (−2.4 kb) and a composite promoter. These enhancers are classified as either early or late based on their developmental activation profile.^33^, ^34^ *LYZ* gene is regulated by a distal upstream cell‐specific enhancer element located 6.1 kb from the transcription start site which is detectable only in cells and tissues where the *LYZ* gene is transcriptionally, and is also controlled by oviduct steroid hormones.^35^ The promoter region of the *LYZ* gene contains binding sites for steroid‐regulated receptors.^36^ The enhancer at this site can bind C/EBP‐like transcription factors, and this interaction is likely primarily mediated by NF‐M, a myeloid‐specific C/EBPβ‐related transcription factor.^37^ The transcriptional activation of the *LYZ* gene locus requires the interaction between enhancers and promoters.^38^

In mouse macrophages, the 3′ flanking region of the *LYZ* gene contains a potent enhancer that is governed by multiple transcription factors and whose activity is modulated by cytosine methylation within its core sequence.^31^ In zebrafish, the myeloid transcription factors C/ebp1, Runx1 and Pu.1, which bind to the promoter region, collaboratively regulate LYZ transcription.^39^ In addition, the c‐Myb transcription factor can also promote LYZ expression by binding to the Cytosine‐Adenine‐Adenine (CAA) motif within its promoter region.^40^

#### 1.2.2 Post‐transcriptional and functional regulation by metabolites, proteins, hormones and cytokines

Beyond control by genetic elements, LYZ expression is modulated by metabolites and other proteins. Lipopolysaccharide mediates the accumulation of LYZ transcripts in the nucleus through enhanced stability of the primary transcript.^41^ Phorbol 12‐myristate 13‐acetate induces LYZ overexpression in airway epithelial cells via the ERK1/2‐c‐Myb signalling pathway.^40^ The myeloid Elf‐1‐like factor can regulate LYZ expression,^42^ and the promyelocytic leukaemia nuclear domain 10 further promotes its expression by interacting with myeloid Elf‐1‐like factor.^43^

In addition, a spectrum of key hormones and cytokines exert regulatory effects on LYZ expression or activity. A 1.8‐fold increase in LYZ activity induced by epinephrine and norepinephrine in vitro is associated with their interaction with the active‐site residues Asp53 and Glu35, which results in reduced activation energy.^44^ The constitutive synthesis and secretion of LYZ is upregulated by IL‐1β, IL‐6 and TNF‐α in a range of hepatocellular carcinoma (HCC) cell lines,^45^ which is related to the activation of STAT3 pathway. In mononuclear macrophages, TLR2 overexpression enhances LYZ expression and secretion, an effect that may be mediated by the concomitant upregulation of IL‐6.^46^

#### 1.2.3 Epigenetic regulation

The regulation of LYZ is further modulated by the epigenetic landscape. During the maturation of human myeloid cells, LYZ expression gradually increases, which is accompanied by progressive demethylation in the gene's 5′ region.^47^, ^48^ In chicken mononuclear macrophages, the LYZ regulatory region in undifferentiated monocytes is marked by monomethylated histone H3 lysine 4 (H3K4me1), which gradually transitions to a trimethylated state (H3K4me3) during cell differentiation. During this process, the LYZ promoter undergoes extensive, differentiation‐dependent alterations in nuclease accessibility.^49^

In addition to histone methylation, alterations in the histone acetylation landscape also significantly influence LYZ expression. In myeloblasts, LYZ expression is low, with only H4 acetylation present at cis‐regulatory elements. In contrast, mature macrophages exhibit high LYZ expression, accompanied by acetylation of H4, H3, H2B and H2A.Z at these regulatory sites. The developmental activation of the *LYZ* gene is linked to the acetylation of core histones at its enhancer elements.^50^

Furthermore, in macrophages, LYZ expression can be rapidly induced by pro‐inflammatory stimuli. This process involves the disengagement of the negative regulatory element from the repressor protein CTCF, the concomitant activation of enhancer elements, and the transient synthesis of a long non‐coding RNA. The production of long non‐coding RNA, in turn, initiates a cascade of epigenetic modifications conducive to LYZ expression, including histone H3 phosphoacetylation across the transcribed region and nucleosome repositioning at the CTCF binding site. This represents a transcription‐dependent chromatin remodelling mechanism that switches the cis‐regulatory landscape of the *LYZ* gene from a repressive to an active conformation.^51^ In summary, LYZ expression is stringently regulated by epigenetic mechanisms.

#### 1.2.4 Insights derived from genetic manipulation of LYZ

Gene editing is paramount for achieving specificity and accuracy in functional genomic studies. Genetic editing of LYZ, particularly through knockout approaches, provides a robust method to further validate the reliability of its observed effects.

Research involving genetic editing of LYZ has predominantly focused on its roles in bacterial killing and immunomodulation. In an early transgenic mouse study, researchers generated mice expressing rat LYZ cDNA specifically in pulmonary respiratory epithelial cells.^52^ These transgenic mice demonstrated a significantly enhanced bactericidal capacity, exhibiting greater than two‐ and five ‐ fold increases in the killing of *Streptococcus* species and *Pseudomonas aeruginosa*, respectively.^52^ Furthermore, this conferred a marked improvement in overall survival. To further validate these findings, subsequent investigations employed a knockout mouse model targeting the murine *LYZ* gene. Conventionally housed LYZ M‐deficient mice exhibited normal morphology and overall health.^53^ Compared to their wild‐type littermates, LYZ M‐deficient mice exhibited a significantly diminished capacity to clear *P. aeruginosa*,^53^ *Micrococcus luteus*,^54^ and *S. pneumoniae*.^55^ Concurrently, LYZ M‐deficient mice developed excessive inflammatory responses,^54^ which exacerbated the pathology induced by bacterial infection.^55^ In a tracheal *Klebsiella pneumoniae* infection model, LYZ M‐deficient mice exhibited a significantly reduced ability to kill the pathogen, which was accompanied by markedly high mortality.^56^ Additionally, CRISPR/Cas9 knockout of LYZ in the human retinal pigment epithelial cell line ARPE‐19 led to a marked decline in bactericidal efficacy and was accompanied by a dampened response to lipopolysaccharide.^57^ These studies, utilizing both knockout and overexpression mouse models or cell lines, have conclusively demonstrated the essential role of LYZ in host bactericidal function and immune responsiveness.

Beyond genetic manipulation studies focused on LYZ's canonical functions, sporadic investigations have also explored its roles in other domains, such as cardiac pathology and oncology. In myocardial infarction, cardiac endothelial cells aberrantly express LYZ 2 (the mouse homolog of LYZ), which promotes pathological degradation of the extracellular matrix by enhancing lysosomal degradative capacity, thereby accelerating disease progression. The deletion of LYZ 2 in mice effectively reverses disease progression and facilitates cardiac regeneration.^8^ In studies on HCC, aberrant expression of LYZ in HCC cell lines has been found to promote cell proliferation and migration. Conversely, knockout of LYZ in these cell lines significantly inhibits cell proliferation, migration, and tumourigenic capacity.^58^ These studies have collectively validated the non‐canonical functions of LYZ in both cardiac regeneration and tumour progression by employing LYZ deletion strategies in mouse models or cell systems, respectively.

#### 1.2.5 Summary of LYZ regulatory networks

To summarize, LYZ is subject to robust regulation by a multifaceted network encompassing genetic elements, transcription factors, metabolites, hormones, cytokines and epigenetic modifications. This regulatory pattern underscores the complexity and diversity of LYZ regulation, and implies that LYZ may be highly susceptible to perturbations from multiple factors. Understanding these established regulatory mechanisms holds significant implications for future interventions targeting cellular dysfunction and even carcinogenesis triggered by dysregulated LYZ expression.

### 1.3 LYZ in cancers

Notably, LYZ's non‐antimicrobial roles, particularly its intricate relationship with cancer, remain underexplored. Early studies hinted at its involvement in tumourigenesis^59^, ^60^, ^61^, ^62^ (Figure 1D), yet the core paradox in this field persists: LYZ inhibits tumour growth and metastasis in some cancers (e.g. breast and lung cancer) while promoting malignancy in others (e.g. HCC and leukaemia). Whether this duality stems from experimental biases or inherent tumour‐type specificity remains unclear.

Advances in multi‐omics technologies – including transcriptomics and proteomics – have revolutionized our ability to systematically observe and evaluate the correlations between LYZ and the development of various tumours, making it possible to unravel these complexities. Focusing on mammalian C‐type LYZ, this review addresses three key questions: (1) What are the mechanistic underpinnings of LYZ's tumour‐suppressive and tumour‐promoting effects? (2) How do multi‐omics data illuminate the cancer‐type specificity of these opposing roles? (3) What is the clinical potential of LYZ as a diagnostic/prognostic biomarker or therapeutic target? By integrating multi‐omics analyses, we aim to clarify LYZ's mechanistic duality, synthesize insights into its context‐dependent functions, and provide a framework to guide future research in oncology.

## 2 THE DUAL ROLES OF LYZ IN TUMOUR PROGRESSION

### 2.1 Tumour suppressive effects

Why has LYZ been investigated as a potential tumour suppressor molecule? Clues can be found through an in‐depth review of previously published literature. The earliest research originated from the observation that bacteria and bacterial degradation products (such as peptidoglycan) could enhance host non‐specific resistance, thereby modulating tumour growth.^63^, ^64^, ^65^, ^66^, ^67^ Specifically, one study reported that a bacterially derived muramyl dipeptide activates macrophages, which in turn initiates a lymphocyte‐mediated immune response that suppresses tumour metastasis.^67^, ^68^ These reports suggest that bacterially derived components can control tumour progression by activating the host immune system, including engaging key immune cells such as lymphocytes and macrophages. Indeed, treatment of bacteria with LYZ precisely generates these immunomodulatory bacterial fragments and metabolites.^67^, ^69^ This effectively establishes the connection between LYZ and tumour suppression. Multiple studies on lung cancer,^67^, ^70^ fibrosarcoma,^71^ melanoma,^72^, ^73^, ^74^ bladder cancer^72^ and breast cancer have been conducted based on this rationale. These investigations have utilized various mouse tumour models – including metastatic, subcutaneous and orthotopic models – to systematically evaluate the tumour‐suppressive effects of LYZ. Based on the potential tumour‐suppressive capability of LYZ, other research efforts have focused on modifying the LYZ protein through various strategies to enhance its anti‐tumour efficacy. Consequently, this has spurred a series of studies focusing on the modification of the LYZ protein, including investigations into stable self‐assembled nanostructured LYZ (snLYZ),^75^ polyoxyethylene glycol (PEG)‐chemical recombinant LYZ^76^ and LYZ conjugated with monomethoxypolyethylene glycol (mPEG‐Lyso).^77^, ^78^ Furthermore, studies on clinical tumour samples utilizing immunohistochemistry (IHC) and newly emerging approaches have revealed aberrant alterations in LYZ abundance within tumour tissues, such as an investigation of a breast cancer cohort.^79^ Driven by the multifaceted rationale outlined above, research on the tumour‐suppressive functions of LYZ has flourished.

**FIGURE 3:**
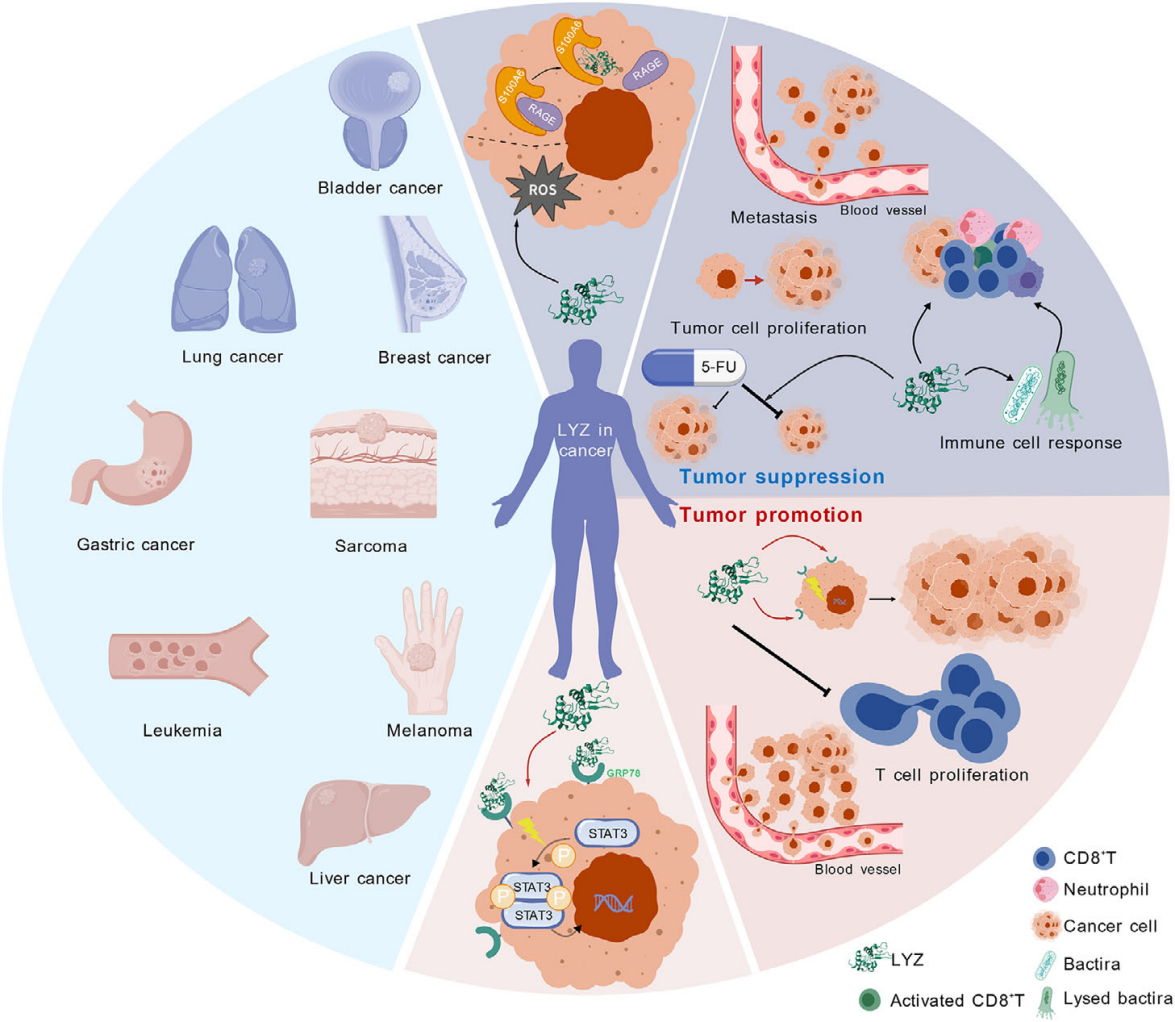
Lysozyme (LYZ) functions in tumour progression and the underlying mechanisms. LYZ has been reported to corelate with the progression of different tumours which are displayed in the left blue area. In breast cancer, lung cancer and bladder cancer, high expression of LYZ is related to favourable clinical outcomes. However, high expression of LYZ correlates with poor clinical outcomes in leukaemia, gastric cancer, sarcoma (such as oral squamous cell carcinoma and granulocytic sarcoma of the eye orbit), melanoma and hepatocellular carcinoma (HCC). The underlying mechanisms through which LYZ affects tumour progression are outlined in the right area. As a tumour suppressor, reported in lung and breast tumours, LYZ inhibits tumour progression by suppressing tumour cell proliferation and metastasis and sensitizing tumour cells to chemotherapy. Specifically, LYZ induces tumour cell death through membrane rupture by promoting the intracellular accumulation of reactive oxygen species (ROS). Concurrently, it inhibits cell proliferation by blocking the interaction between S100A6 and RAGE. It can also trigger immune cell activation through peptidoglycan and polyribopyrimidinic acids associated with the bacterial‐killing process. In contrast, as a tumour driver, reported in liver tumour, LYZ boosts tumour progression by directly promoting tumour cell proliferation and curbing the proliferation of tumour‐infiltrating immune cells. Specifically, LYZ can activate tumour‐promoting pathways, such as the STAT3 signalling pathway, by engaging with GRP78 on the surface of tumour cells. This figure was generated using the Generic Diagramming Platform‐BioGDP.^9^

Extensive research has demonstrated that LYZ exhibits significant anti‐tumour activity across various experimental models, including multiple cell lines and animal systems, with preliminary evidence of therapeutic potential emerging from limited clinical trials in human subjects (Table 1). Most of these studies have primarily focused on its inhibitory effects on tumour metastasis, with robust evidence in several cancer types (Figure 3).

**TABLE 1:** Tumour suppression studies of lysozyme (LYZ).

Tumour type	Study subjects	Cell lines	Location of the tumour	Type of LYZ	Administration method	Dosage	Mechanisms	Conclusions	Limitations	Ref.
Lung cancer	Mouse	Lewis lung carcinoma M1087	Subcutaneous injection	Egg white LYZ	Oral	25–200 mg/kg/day; 3 weeks	Likely mediated by host responses	50%–75% weight reduction of pulmonary metastatic tumours	Lack of mechanistic findings	^67^
Lung cancer	Mouse	Lewis lung carcinoma M1087	Subcutaneous injection	Egg white LYZ	Oral	35 mg/kg/day; 1 week	Probably mediated by the elicitation of host responses	Obviously delaying of pulmonary metastases for more than 2 weeks	Lack of mechanistic findings	^67^
Lung cancer	Mouse	Lewis lung carcinoma M1087	Subcutaneous injection	LYZ chloride	Oral	100 mg/kg/day	Splenic activation & macrophage‐derived multinuclear giant cell induction	Significantly reducing the number of metastatic tumours and the occurrence of lung metastases	Mechanistic investigations lack sufficient experimental support	^70^
Breast cancer	Cell line	MCF‐7	–	Nano‐assembled LYZ	–	200 µg/mL; 24 h	Reactive oxygen species (ROS) accumulation and membrane damage	Killing ∼90% of the tumour cells	Lack of in vivo efficacy validation	^75^
Breast cancer	Mouse	Murine MCa line	Subcutaneous injection	Polyoxyethylene glycolyzed LYZ	After implantation; oral	25–100 mg/kg/day; 14 days	–	Significantly inhibiting primary tumour and spontaneous lung metastasis	Lack of relevant mechanistic investigation	^76^
Breast cancer	Mouse	–	Subcutaneous injection	Egg white LYZ	Oral	100 mg/kg/day; 8 days	Promoting the proliferation of lymphocytes and monocytes	Potentiated the anti‐tumour and anti‐metastatic efficacy of 5‐FU	Mechanistic conclusions lack adequate experimental substantiation	^80^
Fibrosarcoma	Mouse	3‐Methylcholanthrene‐induced sarcoma cells	Subcutaneous injection	Human LYZ	Before implantation; Co‐incubation with cells	150–1000 µg/mL; 18 h	Enhancing tumour cell immunogenicity	Pre‐coincubation protects nearly half of the mice from tumours	Lack of validation for the proposed mechanisms	^71^
Melanoma	Mouse	B‐16 cell line overexpressing LYZ	Subcutaneous injection	Mouse LYZ	–	–	Suppressing the tumourigenicity of tumour cells and inducing of host responses	The tumourigenic rate is significantly lower than that of the control group	The proposed mechanism remains unvalidated	^74^
Melanoma	Mouse	B‐16 cell line	Subcutaneous injection	Egg white LYZ	Oral	50 mg/kg/day; 7 days	Induction of host immune responses	Significantly reducing the progression of spontaneous lung metastases from melanoma	Mechanistic explanations are ambiguous	^73^
Melanoma/bladder cancer	Cell line	Malme‐3 M /T‐24	–	Human LYZ	–	60 µg/mL; 8–48 h	Augmented monocyte‐mediated tumouricidal activity	Human LYZ has a significant enhancement of monocyte‐mediated cytocidal effects	Absence of in vivo experimental validation	^72^
Gastric cancer	Cell line	MGC803, MKN28, MKN45	–	Human LYZ	–	100–1000 µg/L; 20 h	–	Inhibiting the growth of gastric cancer cell lines	Absence of in vivo validation and mechanistic elucidation	^83^
Reticulosarcoma of the small intestine	Human	–	–	Human LYZ	Injection	45 g of LYZ injected at 3 months	Inducing of host responses	Effective elimination of abdominal metastases; high postoperative survival rate of patients	Limited patient cohort	^89^
Myeloma	Human	–	–	Human LYZ	Oral	2 g/day	Inducing of host responses	Combination with chemotherapy can enhance monocyte function	Limited patient cohort	^89^

#### 2.1.1 Lung cancer

In some mouse models of lung cancer, oral administration of LYZ and its derivatives (e.g. LYZ chloride) have shown marked efficacy in Lewis lung carcinoma. In orthotopic and metastatic models established using mouse lung cancer cell lines, LYZ inhibited the development of metastatic solid tumours^67^ and increased the proportion of nonmetastatic and low‐metastatic mice.^70^ Notably, pre‐administration of LYZ 1 week before inducing lung metastasis delayed the onset of metastasis by over 2 weeks,^67^ suggesting potential in preventive applications. Mechanistically, oral administration of LYZ may modulate the spleen cell activation and induction of immune cells (e.g. multinuclear giant cells), which correlates with its anticancer function,^70^ supporting its potential as a therapeutic agent for human lung carcinoma. Although these studies provide credible experimental evidence supporting LYZ's role in suppressing lung cancer metastasis, they lack comprehensive mechanistic elucidation. Deepening the mechanistic investigation into how LYZ suppresses lung cancer metastasis is crucial for solidifying the established link between LYZ and the inhibition of tumour metastasis.

#### 2.1.2 Breast cancer

In breast cancer, both in vitro and in vivo studies highlight LYZ's inhibitory effects. Structurally stable snLYZ induced nearly 95% death in MCF‐7 cells within 24 h,^75^ demonstrating direct cytotoxicity. This effect is linked to snLYZ‐induced intracellular reactive oxygen species accumulation. SnLYZ can be internalized by cells to form granules, which subsequently disrupt the cell membrane and initiate the cell death process.^75^ These studies highlight the direct cytotoxic effects of LYZ on breast cancer cells, yet lack validation in animal models. This limitation restricts the translational potential of chemically modified LYZ as a therapeutic agent against breast cancer. In animal models, PEG‐chemical recombinant LYZ significantly inhibited spontaneous lung metastases.^76^ While oral LYZ enhanced the efficacy of 5‐FU on primary tumour growth and lung metastasis in advanced breast cancer as well.^80^ During this process, LYZ reversed the inhibitory effect of 5‐FU on lymphocyte proliferation in the spleens of mice, while simultaneously promoting monocyte proliferation and enhancing host immunity.^80^ Similarly, another LYZ‐modified derivative, mPEG‐Lyso (LYZ coupled with monomethoxypolyethylene glycol), has been found to inhibit both primary tumours and lung metastatic tumours in mice with breast cancer by oral treatment.^77^ mPEG‐Lyso similarly alleviates the tumour‐mediated suppression of lymphocyte proliferation.^77^ mPEG‐lyso also reduces the expression of ICAM‐1 and E‐cadherin in the lung cancer cell line TS/A, thereby suppressing tumour metastasis.^78^ Given that all the aforementioned LYZ variants have lost their enzymatic activity due to chemical modifications, it can be concluded that LYZ suppresses breast cancer progression in an enzyme activity‐independent manner. Chemically modified LYZ exhibits enhanced functional properties, and its tumour‐suppressive efficacy has been validated through animal experimentation. The biological mechanisms underlying the inhibition of breast cancer growth or metastasis by these chemically modified LYZ variants remain inadequately characterized. Most studies attribute the effects to alterations in the host immune system, yet provide limited detailed elucidation. Clinical data from 177 breast cancer patients reinforced these findings: higher LYZ levels were observed in highly/moderately differentiated tumours and smaller, lymph node‐negative tumours, with reduced expression correlating with shorter recurrence‐free survival and overall survival,^79^ underscoring its diagnostic and prognostic value. Single‐cell sequencing analysis of cerebrospinal fluid from breast cancer patients with brain metastases revealed that LYZ may enhance anti‐tumour immune function by potentiating T‐cell activation and augmenting the antigen‐presenting capacity of antigen‐presenting cells.^81^ Another study utilizing publicly available breast cancer datasets from The Cancer Genome Atlas (TCGA), the Molecular Taxonomy of Breast Cancer International Consortium (METABRIC) and the Gene Expression Omnibus (GEO) revealed that LYZ is highly expressed in tumours with abundant immune cell infiltration.^82^ These studies, utilizing both conventional clinical assays and high‐throughput sequencing on clinical samples from breast cancer patients, demonstrate that elevated LYZ expression can inhibit breast cancer progression. However, the conclusions drawn from these studies are inadequately supported by robust experimental data and lack validation in independent patient cohorts. Collectively, these studies support the notion that LYZ suppresses breast cancer progression by modulating the tumour immune microenvironment (TME).

#### 2.1.3 Other cancers

Evidence for LYZ's tumour‐suppressive role extends to other malignancies. In fibrosarcoma, for example, LYZ can protect model mice from tumours by enhancing the immunogenicity of fibrosarcoma cells.^71^ Using tumour cells pre‐incubated with LYZ in a mouse model of fibrosarcoma significantly reduced the tumourigenicity of mice.^71^ LYZ treatment enhances the immunogenicity of tumour cells, facilitating their recognition by immune cells, and this effect is closely associated with its enzymatic activity.^71^ In melanoma and bladder cancer, LYZ augmented monocyte‐mediated tumouricidal activity, linked to increased metabolic capacity and its enzymatic activity.^72^ LYZ enhances monocyte‐mediated tumour cell killing through its intrinsic cationic properties and by augmenting cellular metabolic activity. For melanoma specifically, oral administration of LYZ analogues reduced spontaneous lung metastases in mice bearing B16 melanoma,^73^ and the LYZ‐overexpressing melanoma cell lines showed diminished tumourigenicity.^74^ Although these two studies lack direct experimental evidence, they support the perspective that LYZ inhibits tumour growth by enhancing host immunity. Additionally, recombinant human LYZ inhibited gastric cancer cell lines at 100 mg/L^83^ and blocked SW480 cell proliferation by disrupting the interaction between S100A6 and the RAGE V domain.^84^ These studies discussed above significantly expand the evidence supporting LYZ's role in inhibiting the growth and metastasis of diverse tumour types. However, these studies employed a limited range of tumour cell types or animal tumour models. More comprehensive experimental data are needed to robustly support the conclusions. Recently, an analysis of published multiple myeloma data from the GEO and TCGA databases revealed that LYZ expression is downregulated in tumours and positively correlates with favourable patient prognosis.^85^ Moreover, LYZ in combination with other genes can be used to construct stable prognostic risk prediction models.^85^, ^86^ Similar methods have been applied in studies of diffuse large B‐cell lymphoma, revealing that high LYZ expression in tumours is associated with favourable patient outcomes and increased immune cell infiltration.^87^, ^88^ In addition, LYZ has also been utilized as a component in constructing robust prognostic risk prediction models.^87^, ^88^ Although the aforementioned studies reveal LYZ's potential as a biomarker for myeloma and diffuse large B‐cell lymphoma, findings derived from public databases require validation through independent patient cohorts.

LYZ may also cause the release of substances responsible for immune enhancement and anti‐tumour activity by bacteria (such as peptidoglycan and polyribosyl pyrimidine acid),^89^ thus activating the immune system. Even more remarkably, during the 1960s, a small number of human trials showed that LYZ effectively inhibited tumours. For example, Laterza injected LYZ into patients with metastatic dissemination in the abdomen after surgery for reticulosarcoma of the small intestine, which was found to eliminate abdominal metastasis.^89^ Despite these observations, the underlying mechanisms remain poorly understood, and updated research is needed to validate these long‐standing discoveries.

### 2.2 The tumour promoting effects

However, why has LYZ also been implicated as a tumour‐promoting factor in some studies? This is primarily attributed to a growing body of research revealing aberrant LYZ expression in various tumour cell types, including HCC,^45^, ^58^, ^90^ gastric carcinoma,^91^, ^92^ oral squamous carcinoma,^93^ male breast cancer,^94^ leukaemia^95^, ^96^, ^97^ and colon cancer.^98^, ^99^ Virtually cells in the normal tissues corresponding to the aforementioned tumour sites show no detectable LYZ expression. This phenomenon strongly implies that the aberrant expression of LYZ in tumour cells actively facilitates carcinogenesis or cancer progression. Furthermore, multiple studies involving IHC analysis of tumour tissues,^94^, ^100^, ^101^ serum tests^102^, ^103^, ^104^ and urine assays^105^, ^106^, ^107^ from cancer patients have consistently revealed elevated expression or aberrantly enhanced activity of LYZ in patient‐derived tumour samples. In addition, other studies utilizing high‐throughput sequencing^108^ or mass spectrometry^58^ have identified abnormally high expression of LYZ in tumour tissues from cancer patients. Based on the induction by the aforementioned multiple factors, a growing number of studies have begun to focus on how LYZ exerts its tumour‐promoting effects.

Meanwhile, extensive studies have highlighted the correlation between LYZ expression and unfavourable clinical outcomes in human subjects (Table 2). Most of these studies have primarily focused on its promoting effects on tumour proliferation and metastasis, with conclusive evidence in several cancer types (Figure 3).

**TABLE 2:** LYZ is a poor prognostic predictor with potential cancer promoting effects.

Tumour type	Sample type	Number of cases	Proportion of positive patients	Detection method	Conclusions	Limitations	Ref.
Gastric cancer	FFPE	83	34.9%	IHC	LYZ may serve as a marker for some gastric cancer.	Lack of validation cohort data	^91^
Gastric cancer	Serum, urine	1	–	ELISA	The elevation of LYZ in serum and urine originated from the secretion of gastric cancer cells.	Single case report with limited generalizability	^92^
Gastric cancer	FFPE	171	38%	IHC	The expression of LYZ predicted poor prognosis in advanced gastric cancer.	Lack of validation cohort data	^109^
Hepatocellular carcinoma	FFPE	21	47.6%	IHC	About half of the HCC samples had positive staining for LYZ.	Limited sample size and absence of a validation cohort	^90^
Oral squamous cell carcinoma	FFPE	30	43.3%	IHC	LYZ is a potential marker of non‐cannibalistic tumour cells of oral squamous cell carcinoma.	Lack of validation cohort data	^93^
Colon cancer	Cell line	–	–	Amino acid sequencing	LYZ was associated with the maintenance of malignancy in colon cancer cell lines.	Lack of investigation into the functional role of LYZ in colon cancer cells	^98^
Male breast cancer	FFPE	60	45%	IHC	The expression of LYZ was associated with poor prognosis in male breast cancer.	Lack of validation cohort data	^94^
Granulocytic sarcoma of the eye orbit	FFPE	7	71%	IHC	The combination of Leder stain and LYZ could be used for the diagnosis of orbital granulocytic sarcoma.	Limited sample size and absence of a validation cohort	^100^
Cutaneous myeloid sarcoma	FFPE	57	91%	IHC	The combination of LYZ, CD117 and CD33 antibodies screened for all tumour patients and 91% of them were positive for LYZ.	Lack of support from an additional validation cohort	^101^
Melanoma/lung cancer	Serum	121/61	–	Turbidimetric method	Tumour patients had significantly higher levels of LYZ than healthy controls.	Lack of validation cohort data	^102^
Leukaemia	Serum, urine	10	–	Agar plate method	High abundance of LYZ in serum and urine is a potential marker for leukaemia.	Limited sample size and Lack of mechanistic investigation	^112^
Glioma	Tumour tissue	1012	–	RNA‐seq	High LYZ expression in tumour tissue is associated with shorter patient survival.	Lack of validation cohort data	^111^

Abbreviations: FFPE, formalin‐fixed, paraffin‐embedded; IHC, immunohistochemistry; LYZ, lysozyme; RNA‐seq; ribonucleic acid sequencing; Ref., references.

#### 2.2.1 Aberrant expression of LYZ in tumour cells

Although LYZ can be widely expressed in various normal organs, cells and body fluids, what is even more surprising is that aberrant expression of LYZ has been found in various tumour cells (Figure 1A). Aberrant expression of LYZ has been reported in tumour cells including HCC,^45^, ^58^, ^90^ gastric carcinoma,^91^, ^92^ oral squamous carcinoma,^93^ male breast cancer,^94^ leukaemia^95^, ^96^, ^97^ and colon cancer.^98^, ^99^ In gastric cancer, immunohistochemical results have shown that LYZ has obvious co‐localization with malignant gastric cancer cells.^91^, ^109^ This retrospective study showed that 38% of gastric cancer pathological tissues contained a high abundance of LYZ, and it emphasized that LYZ with high abundance in tumour tissues is produced by tumour cells.^109^ In oral squamous carcinoma cells^93^ and colon cancer cell lines,^98^, ^99^ LYZ is detected in tumour cells but not in normal cells, implying that high LYZ expression may be correlated with tumour carcinogenesis. And recent single‐cell transcriptomic analysis of metastatic colorectal cancer in mice has identified a subpopulation of LYZ‐positive tumour cells. These cells exhibit Paneth cell‐like characteristics and demonstrate enhanced metastatic potential. Furthermore, these cells display stronger cancer stem cell traits, evidenced by their heightened glycolytic capacity.^108^

**FIGURE 4:**
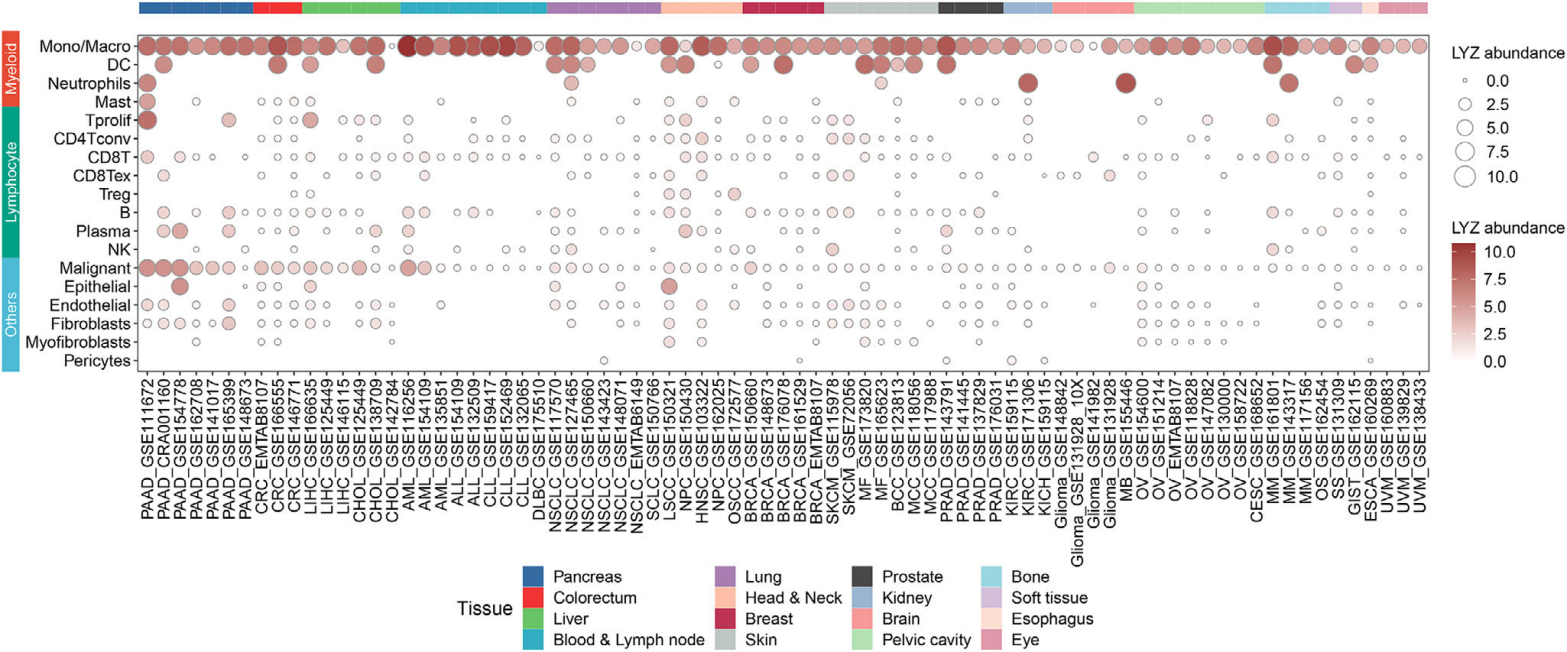
Integrating analysis of lysozyme (LYZ) abundance from single‐cell transcriptomic sequencing data across multiple patient‐derived tumour tissues. According to the published single‐cell transcriptome sequencing data on TISCH (https://tisch.comp‐genomics.org), LYZ expression in different cells was analysed across distinct tumour tissues. Among immune cells, high expression abundance of LYZ is mainly observed in monocytes/macrophages, dendritic cells (DCs), and neutrophils. Unexpectedly, certain malignant tumour cells also show aberrant LYZ expression, especially in PAAD and LIHC. The LYZ abundance is indicated by the size and colour of the bubbles. ALL, acute lymphoblastic leukaemia; BCC, basal cell carcinoma; CHOL, cholangiocarcinoma; CLL, chronic lymphocytic leukaemia; DLBC, lymphoid neoplasm diffuse large B‐cell lymphoma; GIST, gastrointestinal stromal tumour; KICH, kidney chromophobe; LSCC, laryngeal squamous cell carcinoma; MB, medulloblastoma; MCC, Merkel cell carcinoma; MF, mycosis fungoides; MM, multiple myeloma; NPC, nasopharyngeal carcinoma; NSCLC, non–small‐cell lung cancer; OS, osteosarcoma; OSCC, oral squamous cell carcinoma; SCLC, small‐cell lung cancer; SS, synovial sarcoma.

Furthermore, to systematically characterize LYZ expression in the tumour environment across various cancers types, we performed a comprehensive analysis using single‐cell RNA sequencing (scRNA‐seq) data from a public database (Tumor Immune Single‐cell Hub 2 [TISCH2]^110^). Our scRNA‐seq analysis revealed that myeloid cells from all tumours were the predominant cell type that expressing LYZ, with monocytes/macrophages exhibiting the highest expression levels, consistent with previous reports. In addition, tumour cells from various tumours were detected with LYZ expression. The aberrant expression of LYZ by tumour cells in liver hepatocellular carcinoma (LIHC), pancreatic adenocarcinoma (PAAD), acute myeloid leukaemia, and colorectal cancer are particularly prominent (Figure 4). These findings confirm that aberrant LYZ expression by tumour cells is a widespread phenomenon across cancers and strongly suggest a close correlation between such abnormal expression and tumour progression.

#### 2.2.2 LYZ in cancer diagnosis and prognosis

High expression of LYZ in some tumours is closely related to the poor prognosis of patients, suggesting its involvement in promoting tumour progression. In male breast cancer, the survival rate of LYZ‐positive patients is much lower than that of negative patients, indicating that elevated LYZ expression is associated with adverse clinical outcomes.^94^ For localized sarcoma‐orbital granulosa cell sarcoma, Leder staining combined with LYZ and MAC387 immunostaining was discovered to be important in diagnosis.^100^ In a study of cutaneous myeloid sarcoma, a panel including LYZ, CD117 and CD33 was tested across all patients, with 91% showing LYZ positivity.^101^ Furthermore, analysis of serum LYZ levels in patients with some malignant solid tumours revealed significantly higher expression in those with melanoma and lung cancer.^102^ Among melanoma patients at different clinical stages, serum LYZ levels were notably higher in stages II and III compared to stage I, suggesting a positive correlation between LYZ expression and tumour malignancy in melanoma. In glioma research, analysis of transcriptomic data and clinical characteristics from TCGA database revealed that high LYZ expression in tumour tissue is associated with shorter patient survival, indicating its potential as a biomarker for glioma diagnosis and prognosis prediction.^111^ These studies underscore the significant role of aberrant LYZ expression and secretion in facilitating the progression of multiple cancer types, and further emphasize the strong correlation between elevated LYZ levels and unfavourable clinical outcomes in cancer patients. However, to enhance their potential for clinical translation, these findings require further validation through data from corresponding independent patient cohorts to solidify the reliability of the conclusions.

Other studies have also confirmed that serum LYZ levels are significantly higher in patients with untreated malignant melanoma, kidney and breast cancer compared to healthy individuals.^103^ This phenomenon is associated with substantial macrophage infiltration within the tumour tissue.^103^ In recent years, some case reports have also found that overexpression of LYZ by tumour cells have caused renal impairment in leukaemia patients.^95^, ^96^, ^97^ And a large number of LYZ with higher enzyme activity have been detected in leukaemia, which may be beneficial to tumour progression.^104^ More importantly, increased abundance and enzymatic activity of LYZ was consistently observed in both serum and urine samples from nearly all leukaemia patients.^105^, ^106^, ^107^ Another study found a similar phenomenon and concluded that LYZ levels in urine and blood could be used as a potential indicator for the diagnosis of leukaemia.^112^ The above studies together with others have established the status of LYZ as an important biomarker for leukaemia.^113^, ^114^ The studies discussed above collectively emphasize that elevated levels and enhanced activity of LYZ can effectively drive tumour progression and contribute to the manifestation of abnormal physiological parameters in patients. However, the underlying biological mechanisms involved remain to be fully elucidated. Concurrently, these studies necessitate the inclusion of additional independent patient validation cohorts to confirm the stability and reliability of LYZ as a tumour biomarker. It is noteworthy that in patients with chronic myelomonocytic leukaemia, LYZ‐induced nephropathy is one of the most severe complications, significantly accelerating disease progression.^97^, ^115^ These studies demonstrate that LYZ serves as both a tumour‐promoting factor and a potential multi‐cancer biomarker, with significant clinical implications.

#### 2.2.3 LYZ in HCC

Previous studies have discovered that half of HCC samples were found to have positive staining for LYZ by IHC, suggesting that HCC cells can produce LYZ.^90^ For example, HCC cell lines such as HepG2, Hep3B and Huh7 have been observed to express and secrete LYZ constitutively.^45^ And different cytokines can exert distinctly different regulatory effects on LYZ synthesis in these cell lines.^45^ Importantly, HCC cells can promote their own progression through autocrine and paracrine ways independent of LYZ's enzymatic activity, indicating that LYZ plays a significant role in driving HCC progression. Notably, our team's research has further elucidated that LYZ promotes HCC progression by interacting with glucose‐regulated protein 78 (GRP78) on the surface of HCC cells.^58^ This interaction contributes to activating the pro‐tumour signalling pathway of HCC cells, including the STAT3 signalling pathway.^58^ Moreover, the activated STAT3 signalling pathway can positively feedback to promote the expression of LYZ, thus forming a positive feedback loop in HCC cells, which may be crucial for HCC progression. Our study provides a relatively comprehensive and detailed account of the mechanistic basis by which LYZ promotes tumour progression.^58^ However, given the established critical role of immune microenvironment alterations in HCC progression, whether and how LYZ modulates the HCC immune landscape remains an open and insufficiently explored question.

In fact, some studies^116^, ^117^, ^118^, ^119^ have suggested that LYZ may exert its unique functions through cell membrane receptors. Research into LYZ receptor traces back to early studies on the chemosensory adaptation of microorganisms such as paramecia. When LYZ was used as a stimulant, the chemosensory response of effect of paramecia to LYZ was found to depend on a membrane receptor.^116^, ^117^ A similar receptor‐mediated mechanism was subsequently observed in tetrahymena.^117^ Specifically, studies identified distinct LYZ receptor‐like proteins on the membrane surfaces of these microorganisms: a 42 kDa protein in tetrahymena and a 58 kDa protein in paramecia, both of which were newly discovered at the time. Further experiments demonstrated that a neutralizing antibody against 42 kDa protein abrogated LYZ‐induced effect in tetrahymena; this protein, with the characteristic sequence of GGNCSACDAGTSTPAAAQTK, was previously uncharacterized.^118^ LYZ was later confirmed to exert analogous receptor‐dependent effects in paramecia.^119^ These early findings, though derived from microbial models, offer valuable clues for exploring the receptor‐mediated mechanisms of LYZ in cancer.

Based on the above reliable mechanism and phenotypic studies, LYZ neutralizing antibodies and enzyme activity site competitive inhibitors can effectively inhibit the proliferation and migration of HCC cells.^58^ Although enzyme activity site competitive inhibitors of LYZ suppress its interaction with csGRP78 in a concentration‐dependent manner, LYZ promotes HCC progression in an enzyme activity‐independent manner. Targeting LYZ (such as anti‐LYZ antibodies) inhibits HCC progression in both subcutaneous and orthotopic HCC xenograft models. Therefore, LYZ is a promising therapeutic target for HCC. Notably, our prior proteomic study classified HCC patients into three types (S‐I, II, III) based on molecular profiles,^120^ with the most malignant S‐III subtype showing higher LYZ expression in tumour tissues compared to paracancerous tissues. Our subsequent work further revealed that LYZ is significantly overexpressed in poorly differentiated HCC tissues compared to highly differentiated ones, indicating that aberrant LYZ expression in HCC cells correlates with higher malignancy.^58^ Collectively, these findings from our research underscore LYZ's promise as both a prognostic biomarker and a therapeutic target for aggressive HCC.

### 2.3 The modulation of immune cells by LYZ

Indeed, in the context of tumour progression, while the direct impact of LYZ on cancer cells is important, its regulation of various immune cells within the tumour microenvironment (TME) equally deserves considerable attention. Several reviews, based on previous studies, have highlighted that LYZ exerts substantial effects on TME biology by modulating local immune responses.^89^, ^121^ Specifically, LYZ has been reported to target multiple functional cell types in the TME during cancer progression, including lymphocytes, myeloid cells and stromal subsets.^121^

#### 2.3.1 Lymphocytes: Complex regulation of proliferation, function and recruitment

Lymphocytes are central to anti‐tumour immunity, and investigations into the interplay between LYZ and lymphocytes date back to the 1970s.^122^ Early studies established that lymphocytes express surface receptors with specificity for LYZ, enabling recognition of distinct protein epitopes on LYZ^122^, ^123^, ^124^ – laying the groundwork for understanding their functional interactions.

A growing number of studies in vitro identifies LYZ as a potential regulator of lymphocytes proliferation. LYZ is involved in inhibiting the proliferative response of lymphocytes to T cell mitogens, which is closely linked to its enzymatic activity and cationic properties.^125^ Furthermore, LYZ can either enhance or inhibit the proliferative response of human peripheral blood lymphocytes stimulated by interleukin‐2 (IL‐2). This paradoxical effect is contingent upon several factors, including the concentration of IL‐2, the activation status of the cells, the timing of LYZ addition, and the presence of serum. Therefore, LYZ likely modulates lymphocyte proliferation by fine‐tuning their response to antigenic stimuli of specific intensities and regulating the progression of periodic cellular events.^126^ Beyond proliferation, LYZ has also suppresses lymphocyte‐mediated antibody production in a dose‐dependent manner, an effect associated with its enzymatic activity.^127^ This suppression is exacerbated by LYZ‐mediated hydrolysis of peptidoglycan, which amplifies inhibitory signals to lymphocytes.^127^

Notably, tumour progression reshapes this regulatory landscape, as revealed by studies using the MCa mammary carcinomas mouse model. In tumour‐bearing mice, LYZ or its derivative mPEG‐Lyso (which lacks enzymatic activity) restored the proliferative capacity of lymphocytes (e.g. in response to concanavalin A) in the spleens^80^ or the mesenteric lymph nodes.^77^, ^128^, ^129^ Given that mPEG‐Lyso has completely lost its enzymatic activity, this function is likely independent of LYZ's catalytic capability. Additionally, LYZ reinstated the potent pro‐proliferative effect of IL‐2 on lymphocytes,^80^ further supporting its context‐dependent role in lymphocyte activation.

LYZ also modulates lymphocyte recruitment in this model: LYZ treatment increased the number of lymphocytes expressing CD3, CD4, CD8 and CD25 in the mesenteric lymph nodes^129^ with a concomitant increase in the number of lymphatic nodules in gut epithelium.^128^ The increase in CD8^+^ lymphocyte numbers was markedly greater relative to their CD4^+^ counterparts^129^ – an observation with implications for anti‐tumour immunity, as CD8^+^ T cells are key mediators of tumour cell killing.

Clinical evidence further supports LYZ's regulatory role in human lymphocytes: in a trial of patients with malignant tumours, LYZ treatment led to disease remission in the majority of patients (19/21).^130^ Pretreatment abnormalities – including total lymphopenia and a reduced CD4^+^/CD8^+^ ratio were reversed post‐therapy. LYZ therapy demonstrated favourable tolerability and high treatment compliance. Furthermore, studies have demonstrated that LYZ can enhance lymphocyte recognition of tumour cells through surface modification, and this process is dependent on its enzymatic activity.^71^, ^131^ Collectively, these studies confirm LYZ modulates lymphocyte biology (proliferation, function, recruitment) through both enzymatic activity‐dependent and ‐independent pathways. These studies offer initial characterizations of how LYZ influences the functionality and recruitment of major lymphocyte subsets. However, they do not report on the effects of LYZ on functionally critical CD4^+^ and CD8^+^ T cell subsets – such as regulatory T cells (Tregs) and exhausted T cells – likely because most of these studies were completed at an earlier time when these advanced cellular classifications were not fully established or routinely investigated. Indeed, these pivotal cellular subsets are critically important for tumour progression. The impact of LYZ on these critical cellular subsets remains poorly understood.

#### 2.3.2 Myeloid cells: LYZ as a marker and modulator of tumourigenic subsets

Myeloid cells are one of the primary sources of LYZ in the TME. Given LYZ is one of the characteristic markers of myeloid cells,^132^ elevated LYZ abundance in the TME often indicates increased recruitment of myeloid subsets (e.g. macrophages, neutrophils, myeloid‐derived suppressor cells). During tumour development, LYZ actively modulates myeloid cell function to influence tumour progression.

In a Lewis lung carcinoma mouse model, continuous LYZ treatment induced an increase in the population of pulmonary peroxidase‐positive cells. This population comprised not only neutrophils but also newly recruited circulating monocytes.^133^ In the LYZ‐treated group of this model, the reduction in tumour metastasis was concomitant with a significant increase in the number of multinucleated giant cells of monocyte/macrophage origin in the mouse spleen. This suggests that the observed immune activation in mice may be associated with the induction of multinucleated giant cells.^70^

Clinical and preclinical studies further link elevated LYZ to myeloid cell infiltration across cancers: LYZ content or activity is significantly increased in the TME of melanoma,^103^ HCC,^134^, ^135^, ^136^ sarcoma,^137^ pancreatic cancer and breast cancer.^138^ And this elevation is closely associated with myeloid cells such as macrophages. Furthermore, the content and activity of LYZ in the spleen and kidneys gradually returned to normal when anti‐tumour drugs were given to mice subcutaneous seed tumour models of breast cancer, pancreatic cancer and bladder cancer.^138^ It indicates that LYZ that is abnormally expressed by immune cells and tumour cells plays an important role in promoting the progression of malignant tumours, highlighting myeloid cells as both sources and effectors of LYZ's pro‐tumour functions. These early investigations into myeloid cells primarily focused on macrophages, neutrophils and dendritic cells. As research on tumour‐associated myeloid populations deepens, elucidating the effects of LYZ on key subsets – such as M1/M2‐polarized macrophages, myeloid‐derived suppressor cells and tumour‐associated neutrophils – holds significant importance for understanding its regulatory role within the TME.

#### 2.3.3 Enzymatic versus non‐enzymatic roles in immune regulation

The enzymatic activity of LYZ plays a crucial role in immune regulation. The peptidoglycan fragments released through LYZ‐mediated cleavage of bacteria in various tissues or organs (including tumours) serve as important immunoregulatory factors.^139^, ^140^, ^141^ The intracellular receptors NOD1 and NOD2 in monocytes recognize peptidoglycan components and activate downstream pro‐inflammatory signalling by triggering the NF‐κB pathway. This process (LYZ‐mediated peptidoglycan hydrolysis) leads to the secretion of a series of inflammatory factors and is accompanied by immune system activation.^141^ Indeed, numerous studies have demonstrated that LYZ retains immunomodulatory capabilities even in the absence of its enzymatic activity, as clearly evidenced by the mechanisms described previously.^76^, ^77^, ^128^, ^129^ Nevertheless, the role of LYZ in immune regulation especially in cancers still requires further evidence for confirmation.

## 3 LYZ–CANCER RELATIONSHIP FROM A MULTI‐OMICS PERSPECTIVE

**FIGURE 5:**
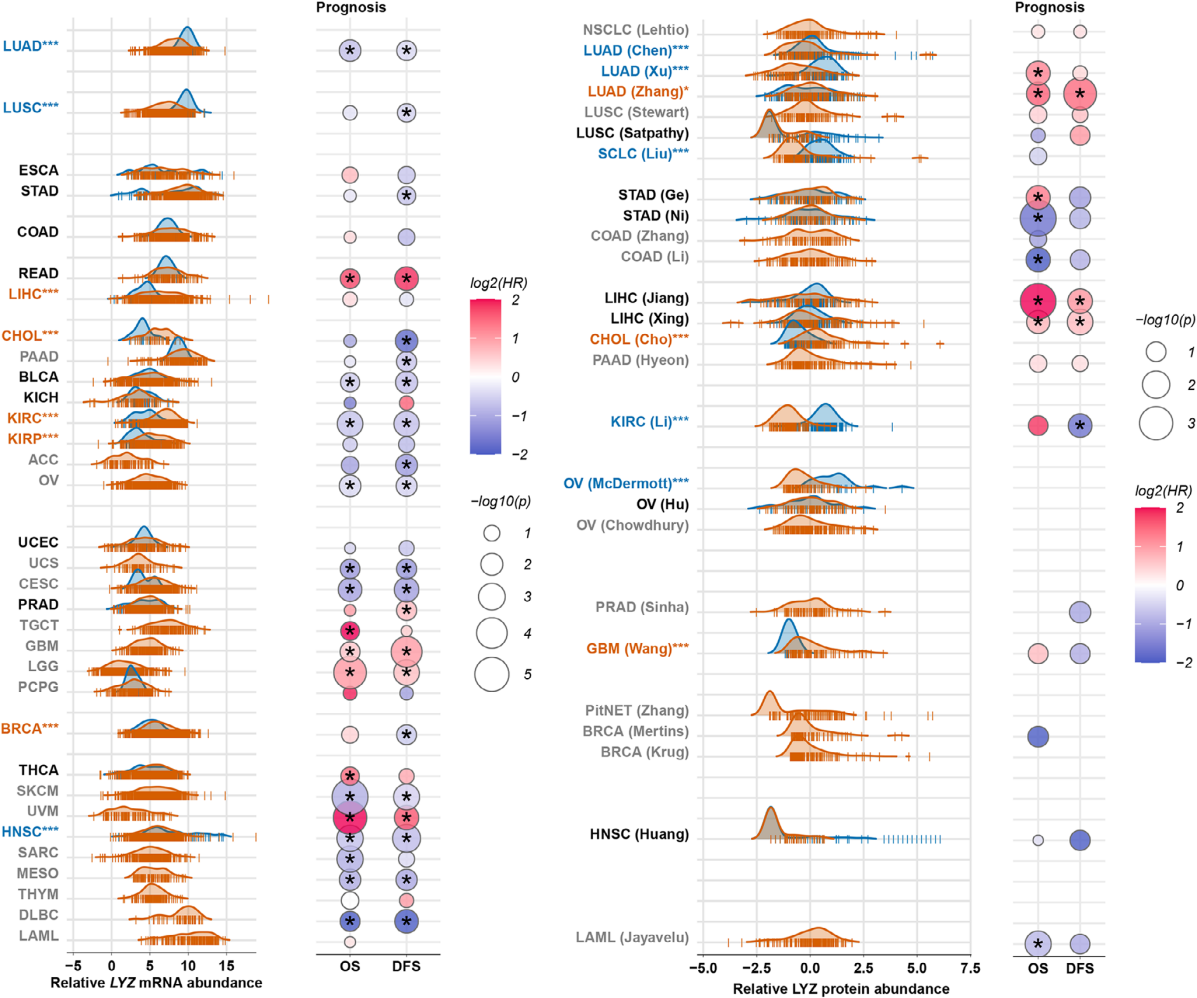
The correlation between lysozyme (LYZ) abundance and patient prognosis in different tumours. By integrating data from the TCGA database, CPTAC database, and additional published sources (NSCLC [Lehtio],^150^ LUAD [Chen],^145^ LUAD [Xu],^146^ LUSC [Stewart],^151^ SCLC [Liu],^152^ STAD [Ge],^153^ STAD [Ni],^154^ COAD [Zhang],^143^ COAD [Li],^155^ LIHC [Jiang],^120^ LIHC [Xing],^149^ CHOL [Cho],^144^ PAAD [Hyeon],^156^ KIRC [Li],^157^ OV [McDermott],^158^ OV [Hu],^159^ OV [Chowdhury],^160^ PRAD [Sinha],^161^ PitNET [Zhang],^162^ BRCA [Krug],^163^ LAML [Jayavelu]^164^), the LYZ expression abundance was analysed and compared between tumour tissues (the brown curve) and paired adjacent noncancerous tissues (the blue curve) in different cancer cohorts. The tumours for which LYZ expression in tumour tissues is statistically higher than that in adjacent noncancerous tissues are indicated in brown font. The tumours for which LYZ expression in adjacent noncancerous tissues is statistically higher than that in tumour tissues are indicated in blue font. The tumours for which there is no statistical difference of LYZ expression between tumour tissues and adjacent noncancerous tissues are indicated in black font. The tumours with unavailable adjacent noncancerous data are indicated in grey font. Meanwhile, the correlation between LYZ expression and patient prognosis (overall survival [OS] and disease‐free survival [DFS]) in different tumours was assessed and displayed with the bubble chart. Different colours denote different HR values of LYZ expression to tumoural prognosis. The statistical difference for HR is shown with bubbles of different sizes, and the difference with statistical significance is marked with a star in the bubble. An HR > 1 (log_2_(HR) > 0) indicates an increased risk of the OS and DFS for patients with high LYZ expression in tumours (unfavourable or poor prognosis), an HR < 1 (log_2_(HR) < 0) suggests a reduced risk (favourable or good prognosis), and an HR = 1 (log_2_(HR) = 0) indicates no difference in risk between the groups. *p <  0.05; ***p <  0.001. Relative LYZ mRNA/protein abundance: The abundance of LYZ in tumour tissue or adjacent noncancerous tissue was normalized and processed using the logarithm with base 2. COAD, colon adenocarcinoma; ESCA, esophageal carcinoma; HR, hazard ratio; PCPG, pheochromocytoma and paraganglioma; PitNET, pituitary neuroendocrine tumour; TGCT, testicular germ cell tumours; THCA, thyroid carcinoma; THYM, thymoma; UCEC, uterine corpus endometrial carcinoma.

Large cohort studies integrating multi‐omics approaches have become pivotal in advancing precise diagnosis and treatment. Here, we utilize transcriptomic and proteomic data to systematically explore LYZ's association with diverse tumours, analysing its expression patterns at both the transcript and protein levels, as well as its clinical value in prognosis and therapeutic guidance. Transcriptomic data were derived from TCGA, and proteomic data from the Clinical Proteomic Tumor Analysis Consortium (CPTAC) and other available published studies (Figure 5).

### 3.1 Transcriptomic profiling: Heterogeneous expression and prognostic associations

TCGA transcriptomic data revealed distinct patterns of LYZ expression and prognostic relevance across cancers. High LYZ mRNA correlated with poor prognosis in uveal melanoma, rectum adenocarcinoma, brain lower grade glioma, glioblastoma and prostate adenocarcinoma (PRAD). Conversely, high expression of LYZ was linked to favourable outcomes in 17 cancer types, including ovarian serous cystadenocarcinoma, bladder urothelial carcinoma, kidney renal clear cell carcinoma, lung adenocarcinoma (LUAD), head and neck squamous cell carcinoma, skin cutaneous melanoma, mesothelioma, cervical squamous cell carcinoma and endocervical adenocarcinoma, uterine carcinosarcoma, breast invasive carcinoma, stomach adenocarcinoma, PAAD, lung squamous cell carcinoma, cholangiocarcinoma (CHOL), adrenocortical carcinoma, lymphoid neoplasm diffuse large B‐cell lymphoma (DLBC) and sarcoma.

Tumour versus paracancerous tissue comparisons further highlighted heterogeneity: LYZ mRNA was upregulated in kidney renal clear cell carcinoma, breast invasive carcinoma and CHOL tumours but downregulated in LUAD, head and neck squamous cell carcinoma, and lung squamous cell carcinoma. Notably, in LIHC and kidney renal papillary cell carcinoma, although the expression of LYZ in tumour tissues was significantly higher than that in paracancerous tissues, it was not significantly correlated with prognoses. However, although the expression of LYZ in PRAD and stomach adenocarcinoma showed no significant difference between tumour tissues and paracancerous tissues, it was correlated with prognoses for these patients. These findings reflect functional heterogeneity of LYZ across cancers and inter‐patient variability within subtypes. However, transcriptomic data alone, which primarily captures mRNA expression, offers limited insights into the actual biological phenotypes, as it fails to account for post‐transcriptional modifications, translational regulation and protein functional states that ultimately drive cellular behaviour.

### 3.2 Transcriptome–proteome discrepancies: Mechanistic underpinnings

Proteomics, as a readout closer to biological function, offers critical insights into LYZ's roles, given proteins are the primary executors of biological activity. Since the National Cancer Institute (NCI)–CPTAC initiated tumour proteomic annotation in 2013^142^ and completed the first proteomic molecular typing of colon and rectal cancer in 2014,^143^ proteomic data have increasingly clarified transcriptomic ambiguities. In this paper, we assess the value of LYZ for clinical applications in the context of published proteomic data from different cancer patient cohorts. In fact, in comparing LYZ abundance in proteomics and transcriptomics of the same cancer, only a few cancers have consistent trend, such as CHOL.^144^ In LUAD, proteomic data from Chen et al.^145^ and Xu et al.^146^ showed higher LYZ in paracancerous tissues, while Zhang et al.’s^147^ cohort yielded opposing results – likely due to differences in patient inclusion criteria (e.g. smoking status, clinical stage). However, all three LUAD cohorts confirmed that high tumour LYZ protein correlated with poor prognosis, aligning with transcriptomic trends. These discrepancies are more likely attributed to post‐transcriptional, translational control and protein degradation regulation rather than technical bias, as exemplified by post‐transcriptional modification and epigenetic regulation.^148^

### 3.3 Subtype‐specific associations: Guiding clinical stratification

**FIGURE 6:**
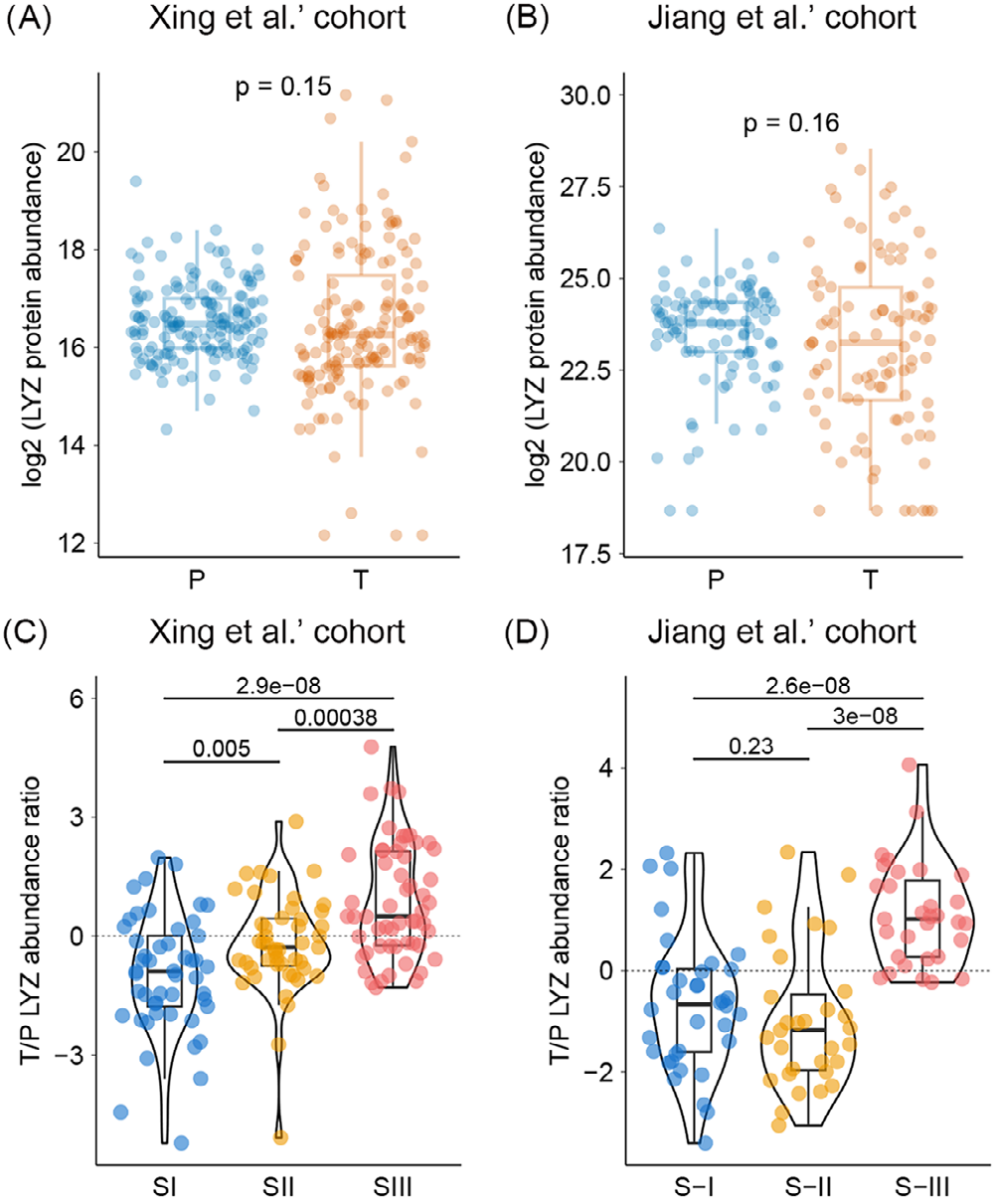
Distinct lysozyme (LYZ) expression in different molecular subtypes of hepatocellular carcinoma (HCC) highlights the significance of molecular typing for the precise tumour diagnosis and treatment. LYZ expression of tumour tissues (T) and adjacent paracancerous tissues (P) in Xing et al.’s^149^ (A) and Jiang et al.’s^119^ (B) HCC cohorts. Relative LYZ expression of tumour tissues to adjacent paracancerous tissues in different molecular subtypes of HCC in Xing et al.’s (C) and Jiang et al.’s (D) HCC cohorts. In both HCC cohorts, the LYZ expression levels were much higher in tumour tissues than that in paired adjacent paracancerous tissues for the S‐III subtype patients, but not for the S‐I/II subtype patients. T/P LYZ abundance ratio: The ratio of LYZ abundance between tumour tissue and adjacent paracancerous tissue was processed using the logarithm with base 2.

To further clarify LYZ's expression and prognostic value across cancer subtypes, taking LIHC as an example: in LIHC, although LYZ abundance in tumour tissue was significantly higher than in paracancerous tissues at the transcriptome level, proteomic data showed no significant difference between Jiang et al.’s^120^ and Xing et al.’s^149^ cohort (Figure 6A,B). Critically, proteomics – unlike transcriptomics – identified prognostic value: high LYZ protein in LIHC tumours predicted poor outcomes in both cohorts. These results also reflect the inconsistency between transcript levels and protein levels. Jiang et al.’s proteomic subtyping of LIHC further uncovered subtype‐specific patterns: only in the aggressive S‐III subtype, tumour LYZ protein was significantly higher than that in paracancerous, and this elevation was associated with shorter disease‐free survival and overall survival^58^; in contrast, LYZ protein levels were decreased in tumour tissues compared to paracancerous tissues in the S‐I/II subtypes (Figure 6C,D). Based on these proteomic insights, targeting LYZ represents a potentially effective therapeutic approach for patients with S‐III subtype HCC. This not only underscores the significant heterogeneity among LIHC patients but also emphasizes the critical importance of precise patient stratification for accurate prognostic prediction. Such findings may enable the implementation of LYZ‐based patient stratification for the high‐risk S‐III subtype of HCC by IHC staining. Therapeutically, S‐III patients with high LYZ expression may benefit from LYZ‐neutralizing antibodies^58^ or STAT3 inhibitors (which disrupt the LYZ‐GRP78‐STAT3 feedback loop), thereby highlighting the value of subtype‐specific targeting strategies.

## 4 PROSPECTS

Through the comprehensive and in‐depth discussion of the current research landscape of LYZ in cancer presented above, we have observed that, on the one hand, the study of LYZ in this field boasts a long history and substantial content, while on the other hand, it reveals numerous limitations inherent in the existing articles. If future research can address the shortcomings of previous studies, it will hold significant importance for clarifying the role of LYZ in cancer.

### 4.1 Interpretability of LYZ's functions in cancer

The role of LYZ in tumours biology remains a topic of ongoing debate. LYZ has been shown to inhibit breast and lung cancers, yet it may also promote the development of gastric and liver cancers. Numerous studies, utilizing cellular and animal models as well as sporadic clinical trials, have provided evidence for both the tumour‐suppressive and tumour‐promoting effects of LYZ. However, a critical issue prevalent in these prior phenotypic studies is the lack of mechanistic explanation. In the past, LYZ was generally thought to be primarily secreted by immune cells but is now known to be abnormally expressed by some tumour cells. Aberrant expression of LYZ has been detected in gastric cancer, HCC and other tumour tissues. Previous studies have often focused on explaining the functions of LYZ in immune cells, such as macrophages, making it challenging to elucidate its roles in tumour cells. Therefore, most studies can only speculate on the role of LYZ in tumours based on some preclinical models and clinical sample from cancer patients, which often leads to confusion in conclusions and low credibility. A portion of the mechanisms reported in the published literature are hypothetical constructs and speculations proposed by researchers based on limited experimental observations. Conclusions related to these mechanisms suffer from a critical shortage of precise experimental data for support.

Therefore, providing precise mechanistic explanations for LYZ's roles in either tumour suppression or promotion is crucial for understanding its overall function in cancer. Firstly, the direct mechanisms by which LYZ acts on tumour cells should receive comprehensive attention, encompassing its effects on processes such as tumour cell proliferation and metastasis. The underlying molecular mechanisms warrant focused investigation. In addition, the effects of LYZ on stromal cells within the TME deserve increased attention, particularly concerning immune cells and fibroblasts, which are closely associated with tumour progression. Simultaneously, the prospective combined application of LYZ or LYZ‐targeting agents with various existing cancer therapies – including chemotherapeutic drugs and immune checkpoint inhibitors – warrants focused consideration. This will facilitate and support the interpretability and potential application of LYZ as either a promising tumour suppressor molecule or a pro‐tumourigenic target.

### 4.2 The regulations of LYZ in the tumour immune microenvironment

The critical role of the immune microenvironment within tumours in driving cancer progression has been extensively demonstrated. As noted earlier, the functions exerted by LYZ on immune cells, particularly lymphocytes and myeloid cells, have been revealed to a certain degree. Nevertheless, these studies present several issues that require resolution. Many studies have been conducted in non‐oncological research contexts. This provides limited insight into how LYZ regulates the immune microenvironment. Furthermore, previous research has predominantly focused on the proportional changes of major immune cell populations within the immune microenvironment, lacking detailed characterization of several critical cellular subsets that are intimately linked to tumour progression. Concurrently, the potential mechanisms through which LYZ‐modulated immune cells contribute to tumour progression are also poorly understood.

Therefore, to advance our understanding of how LYZ modulates the tumour immune microenvironment, future research will likely require multifaceted exploration. Firstly, there is a need to develop more studies investigating the regulation of immune cells by LYZ within tumour systems. Secondly, beyond clarifying the integrated impact of LYZ on broad categories of immune cells such as lymphocytes and myeloid cells, greater attention must be directed towards specific subsets – including exhausted T cells and regulatory T cells within the lymphocyte compartment, as well as M1/M2 polarized tumour‐associated macrophages, tumour‐associated neutrophils among myeloid cells and other immune cell subsets intimately involved in tumour progression. Furthermore, the potential mechanisms by which LYZ regulates multiple processes in these cells – such as their proliferation, differentiation, function and recruitment – should also be a focus of inquiry. Strengthening these research directions is paramount for elucidating LYZ's regulatory role in the TME.

### 4.3 Prospects based on large‐scale cancer patient cohort studies

Many prior studies involving large cohorts of cancer patients, with clinical testing and multi‐omics data, have provided substantial support for the potential clinical utility of LYZ in cancer. These studies also offer valuable references for future research in terms of directions that are amenable to optimization and refinement. Based on IHC or published multi‐omics data from cancer patients, LYZ has been identified as a potential biomarker and shows a significant correlation with patient prognosis across multiple tumour types. These conclusions still require corresponding validation in larger cohorts of cancer patients, which is indispensable for solidifying the clinical application value of LYZ.

For studies involving clinical samples from cancer patients, the significant variations in patient enrolment criteria, detection methods and analytical approaches across different studies have directly contributed to the inconsistency in conclusions. Both earlier IHC results and multi‐omics data indicate that the correlation between LYZ abundance and patient prognosis is quite complex in various tumours. However, there is little agreement between transcriptomic and proteomic data from the same tumour according to the analysed data. These inconsistencies arise from tumour heterogeneity, cohort‐specific variables and post‐transcriptional/translational regulation – underscoring the need for multi‐omics–guided patient stratification. Such an approach has already identified LYZ as a potential biomarker and therapeutic target in S‐III subtype HCC, demonstrating its utility in advancing precision medicine.

Notably, since bulk samples integrate signals from tumour cells, immune cells and stromal cells, high LYZ mRNA/protein levels cannot be directly attributed to either tumour cells or immune cells alone. As demonstrated in Figure 5 using TCGA and CPTAC data, conflating LYZ from different cellular sources may compromise accurate assessment of its functional roles and prognostic value in tumours. This inherent limitation poses a significant challenge for interpreting the functional and prognostic significance of LYZ in bulk data derived from patient cohorts. Two feasible strategies to resolve this issue are proposed: (1) co‐integration of cell‐type‐specific markers in bulk data analysis: by incorporating markers for LYZ‐expressing cell types (e.g. CD68 for macrophages), this approach can correlate LYZ's prognostic value with the infiltration patterns of specific cell populations. For instance, when LYZ levels are combined with CD163 (M2 macrophage marker) expression, high LYZ levels were linked to poor prognosis only when accompanied by increased M2 macrophage infiltration, clarifying that LYZ's prognostic significance here reflects immune microenvironment remodelling rather than tumour cell‐autocrine activity. (2) High‐resolution technological approaches: Single‐cell sequencing technology enables precise detection and characterization of gene expression patterns across distinct cell populations within tumour tissues. Spatial multi‐omics approaches further enhance our understanding by facilitating partitioned analysis of tumour specimens, allowing detailed tracking of molecular abundance variations across functionally distinct regions such as the tumour parenchyma and immune cell–invaded areas. This advancement holds significant implications for delineating the functional and prognostic significance of LYZ derived from distinct cellular origins within the tumour context. In summary, both analytical refinement and advanced technological approaches offer viable pathways to resolve the challenges arising from an inability to distinguish LYZ from different cellular sources in bulk data.

## 5 CONCLUSIONS

LYZ is a multifunctional secreted factor that integrates both antibacterial and immunomodulatory functions. In addition to inhibiting pathogens and participating in immune regulation through various pathways, LYZ can also directly affect tumour cells, modulate the immune microenvironment and play a role in regulating tumour progression. Although more detailed mechanistic studies and clinical investigations are lacking, the current evidence still supports LYZ as a promising potential biomarker and therapeutic target in some tumours. LYZ plays a significant role in tumour diagnosis and therapy, serving as both a potential biomarker for cancer detection and a therapeutic target for some treatment strategies. Its dual functionality, exhibiting tumour‐promoting or tumour‐suppressing effects in different tumours, highlights its complex involvement in cancer biology. Further research into the mechanisms underlying LYZ's actions may pave the way for its clinical application in precision oncology.

## AUTHOR CONTRIBUTIONS

Lei Wang wrote the original draft and contributed to the revision of the manuscript. Qian Dong contributed to the data analysis and drawing of the figures in the manuscript. Fuchu He provided constructive suggestions and guidance on this manuscript. Zhiwen Gu and Aihua Sun critically revised and reviewed the manuscript. All authors contributed to the article and approved the final manuscript.

## CONFLICT OF INTEREST STATEMENT

The authors declare no conflicts of interest.

## Data Availability

The data that support the findings of this study are openly available in TISCH (https://tisch.comp‐genomics.org), TCGA database (https://portal.gdc.cancer.gov/), CPTAC database (https://proteomic.datacommons.cancer.gov/pdc/) and additional published sources.^120^, ^143^, ^144^, ^146^, ^149^, ^150^, ^151^, ^152^, ^153^, ^154^, ^155^, ^156^, ^157^, ^158^, ^159^, ^160^, ^161^, ^162^, ^163^, ^164^
